# First Report of Low Pathogenic Avian Influenza Subtype H9N2 in African Houbara Bustards (*Chlamydotis undulata undulata*) and Gamebirds in Morocco: Clinico-Pathological Findings, Molecular Characterization, and Associated Coinfections

**DOI:** 10.3390/v15122374

**Published:** 2023-12-01

**Authors:** Yassmina Bidoudan, Mohamed Mouahid, Ouafaa Fassi Fihri, Enrico Bollo, Oumayma Arbani, Mariette Ducatez, Brahim Banni, Noursaid Tligui, Siham Fellahi

**Affiliations:** 1 Mouahid’s Veterinary Clinic, Temara 12010, Morocco; mohamedmouahid@gmail.com (M.M.); banni.brahimvet@gmail.com (B.B.); 2 Anatomic Pathology Unit, Department of Veterinary Science and Public Health, Agronomy and Veterinary Institute Hassan II, Rabat 10000, Morocco; n.tligui@iav.ac.ma; 3 Infectious Diseases Unit, Department of Veterinary Science and Public Health, Agronomy and Veterinary Institute Hassan II, Rabat 10000, Morocco; o.fassifihri@iav.ac.ma; 4 Anatomic Pathology Unit, Department of Veterinary Sciences, University of Turin, 10095 Grugliasco, Italy; enrico.bollo@unito.it; 5 Avian Pathology Unit, Department of Veterinary Science and Public Health, Agronomy and Veterinary Institute Hassan II, Rabat 10000, Morocco; arbani.oumayma@gmail.com; 6 Interactions Hôtes-Agents-Pathogènes (IHAP), Toulouse University (ENVT), Institut National de Recherche pour l’Agriculture, l’Alimentation et l’Environnement, 31300 Toulouse, France; mariette.ducatez@envt.fr

**Keywords:** Avian influenza, H9N2, houbara bustards, *Chlamydotis undulata undulata*, gamebird industry, Morocco

## Abstract

We report in this paper the first detection of low pathogenic avian influenza (LPAI) subtype H9N2 in houbara bustards and in gamebirds in Morocco. Starting in 2019, an increase in mortality rates related to respiratory distress was recorded in these species. Necropsy of the specimens revealed fibrinous sinusitis and tracheitis with intra-bronchial fibrin casts, which are consistent with H9N2 infection in chickens; therefore, implication of the virus in these outbreaks was strongly suspected. Consequently, between January 2020 and June 2023, birds with respiratory signs were necropsied for pathological lesions, tissue samples were examined by histopathology, and samples of trachea, lungs, and cecal tonsils were analyzed using quantitative real-time PCR for the detection of H9N2 virus. In addition, the sequencing of isolates was performed and lastly differential diagnosis with other respiratory pathogens was carried out. During the study period, 93 samples were collected from suspected H9N2 outbreaks, of which 30 tested positive for H9N2 virus: 23 Houbara bustards, 4 partridges, 2 quails, and 1 pheasant. Moreover, sequencing of the HA gene of the virus showed 97.33% nucleotide identity with strains reported previously in broilers in Morocco in 2017 and in 2022. Phylogenetic analysis grouped the Moroccan partridge isolates in the same cluster as viruses isolated in Morocco between 2016 and 2022, Algeria (2017), Burkina Faso (2017), Nigeria (2019), and Togo (2020). Additionally, 10 house sparrows from the premises of these birds were examined for the presence of H9N2 virus, revealing a 30% positivity rate. In conclusion, LPAIV H9N2 is circulating in houbara bustards and gamebirds in Morocco, and house sparrows might be a possible source of the infection. To our knowledge, this is the first report of LPAI H9N2 in the African species of houbara bustards worldwide and in gamebirds in Morocco.

## 1. Introduction

The houbara bustard is a medium-sized bird of semi-desert environments belonging to the *Otididae* family [1,2]. The African species (*Chlamydotis undulata undulata*) consists of sedentary populations that occupy arid regions extending from the Canary islands to Morocco, and Egypt [1,3] while the Asian species (*C. undulata macqueenii*) is migratory and occupies regions from the Middle East to Central Asia [2,3]. Both species are listed as vulnerable on the International Union for the Conservation of Nature’s (IUCN) red list [3]. This is due to the over-exploitation of the birds and their natural habitat [1,2,3,4]. As a consequence of the population decline and due to their major role in Arab falconry as the traditional quarry for falcons, numerous conservation sites based on captive breeding and release in nature programs have been established in Morocco since the 1990s by Arab falconers [1]. Likewise, the gamebird industry has recently grown in Morocco, currently comprising twelve centers with a population of 420 100 birds. The most common species of gamebird is the Barbary partridge (*Alectoris barbara*), which registered a 27% increase in its population compared to the year 2021, followed by ring-necked pheasants (*Phasianus colchicus*) and Japanese quails (*Cortunix Japonica*). The centers are distributed in nine regions of the country, with the most significant populations located in Safi and Bouznika, which host 74% of the total gamebird population in the country (Agence Nationale des Eaux et Forêts, 2022). In those conservation sites, captive-bred bustards are released to aid in the restoration of wild populations, while captive-bred gamebirds are released for hunting purposes. This dynamic creates an ideal environment for the spread and persistence of infectious pathogens between the two populations [5]. As a result, active surveillance of infectious diseases must be carried out regularly to maintain the success of both the restoration of bird populations and the health status of the captive and wild populations. Avian influenza viruses (AIV) and Newcastle disease virus (NDV) ought to be monitored by virtue of their important economic, epidemiological, and pathological roles in avian species worldwide [6].

The low pathogenic avian influenza subtype H9N2 has been endemic in intensive poultry farming in Morocco since its introduction in 2016. Reports of the virus circulating in intensive farming units of chickens (broilers, broiler breeders, and layers) and in turkeys have been made [7,8,9,10]. Furthermore, El Mellouli et al. [11] have conducted a prevalence study of H9N2 in wild birds using real time RT-PCR. The study revealed the circulation of LPAI H9N2 in ten bird species belonging to three orders including *Charadriiformes*, *Pelecaniformes*, and *Gruiformes*. However, the prevalence and pathogenicity of the virus in minor reared species including gamebirds and bustards have not yet been studied, although they are thought to play a major epidemiological role in avian disease persistence and circulation. In fact, several studies performed on gamebirds infected with AIV reported their important role as bridge species in the poultry–wildlife interface [10,12,13,14].

In this article, we report for the first time the circulation of the virus in houbara bustards and gamebirds in Morocco. A comprehensive bacteriological study of the major coinfecting agents associated with the infection and the pathogenicity of the virus are also described. In addition, house sparrows (*Passer domesticus*) sampled from the same regions with reported outbreaks were tested using RT-qPCR in an attempt to identify the origin of the virus in these outbreaks. In fact, a risk analysis study was carried out to identify the major routes of exposure of captive-bred houbara bustards to avian influenza viruses (AIV) and Newcastle disease virus (NDV) in the UAE [6]. The authors reported the highest risk associated with house sparrows either via direct contact with the birds or via indirect contact through their visiting bustard aviaries for water and food, resulting in contamination of the water with feces.

## 2. Materials and Methods

### 2.1. Bird Species and Specimen Collection

From January 2020 to June 2023, a total of 93 cases with respiratory syndromes were submitted to our veterinary clinic (Temara, Morocco) and to the avian pathology unit of Hassan II’s Veterinary and Agronomy Institute (Rabat, Morocco) for laboratory investigations. Among these cases, 69 consisted of both vaccinated and non-vaccinated houbara bustards (*Chlamydotis undulata undulata*), 4 were from flocks of ring-necked pheasants (*Phasianus colchicus*), 13 were from flocks of Barbary partridges (*Alectoris Barbara*), and 3 were from flocks of Japanese quails (*Cortunix Japonica*). All the flocks of gamebirds were vaccinated against the LPAI H9N2 virus. The samples of the houbara bustards originated from the southern regions of Morocco, while the gamebirds were from three regions including Tangier–Tetouan–Al Hoceïma, Casablanca–Settat, and Rabat–Sale–Kenitra. In addition, ten house sparrows (*Passer domesticus*) collected from the premises of houbara bustards were examined as described below for the other bird species.

During each H9N2 suspected outbreak, samples of trachea, lungs, and cecal tonsils were collected and stored at −20 °C until processing. Differential diagnosis with other respiratory viral, bacterial, and fungal pathogens was carried using species-specific PCRs with Kylt^®^ Kits (AniCon Labor GmbH, Hoeltinghausen, Germany), namely, *Mycoplasma Gallisepticum* (MG), Paramyxovirus type 1 (NDV), *Chlamydia Psittaci*, infectious laryngotracheitis virus (ILT) and Avian Metapneumovirus (AMPV), in addition to avian coronavirus as part of a monitoring program. Moreover, bacteriology and mycology investigations were also performed according to standard procedures to rule out an *Aspergillus* sp. infection, and to identify bacterial coinfecting pathogens. Finally, scrapings of the crop and the intestines were examined for parasitic helminthes and protozoans.

### 2.2. Sample Processing

#### 2.2.1. Pathological Examination

The dead birds presented to the clinic were necropsied for gross pathologic lesions, and samples of the trachea, lungs, air sacs, kidneys, and pancreas were taken and placed in a 10% solution of neutral buffered formalin for histopathology examination. The samples were then dehydrated and embedded in paraffin and sections of 5 μm were prepared and stained with hematoxylin and eosin (H&E) according to standard histopathologic procedures [15].

#### 2.2.2. RNA Extraction and Real-Time RT-PCR

Viral RNA was extracted from samples of the trachea and lungs (two to five organs pooled per flock) with a viral RNA extraction kit (Kylt, Anicon, Germany), according to the manufacturer’s instructions. The Superscript III-based one-step RT-PCR kit (Thermo Fisher Scientific, Waltham, MA, USA) was used in a TaqMan real-time RT-PCR assay and performed with the primers and probe described by [16] for the amplification of influenza H9 viral nucleic acids in a Fast RT-PCR machine (ABI 7500). For N2 subtyping, a pair of primers specific for the N2 subtype was used for PCR amplification [17]. The cycling conditions consisted of 30 min at 50 °C (reverse transcription phase) and then an initial denaturation at 95 °C for 2 min, followed by five touchdown PCR cycles starting with 94 °C for 15 s, 60 °C for 30 s, 68 °C for 1 min; 30 cycles of 94 °C for 15 s, 54 °C for 15 s, 68 °C for 1 min, and a final extension at 68 °C for 5 min, according to the manufacturer’s instructions.

#### 2.2.3. Virus Isolation

Samples with the highest Ct values in the RT-PCR were grown in 10-day-old specific pathogen-free (SPF) embryonated chicken eggs in order to obtain a maximum viral load detectable by conventional RT-PCR. The viral inoculum was prepared as described previously by Sikht et al. [10] and then injected into the air chamber. The eggs were incubated at 37 °C and the embryos’ viability was evaluated daily. Upon the embryos’ death, the eggs were refrigerated at 4 °C for 4 h, after which lesions on the embryos were observed and the allantoic fluids were collected, clarified, and stored at −80 °C until use.

#### 2.2.4. HA Gene Sequencing

Amplification of the partial HA gene by RT-PCR was carried out using the primers described by Hoffmann et al. [18]. The RT-PCR was achieved using the Applied Biosystems kit (Life Technologies). The RT-PCR reaction was performed in a 20 μL reaction mixture containing 2 μL of buffer (10×), 2.5 μL of MgCl_2_ (25 mmol/L), 2.5 μL of dNTP (10 mmol/L), 0.75 μL of each primer (10 μmol/L), 10.2 μL of sterile water, 0.5 μL of RNAase inhibitor (20 U/μL), 0.3 μL of RT (50 U/μL), and 0.5 μL of Gold Taq polymerase (5 U/μL). Forty cycles at 94 °C for 20 s, 56 °C for 20 s, and 72 °C for 30 s were carried out. A final step at 72 °C for 2 min was added to complete amplification. The PCR products were analyzed on a 1% agarose gel. RT-PCR products (500 bp) containing a region known to vary between strains were purified with the Nucleospin gel and a PCR cleanup kit (Macherey Nagel,Düren, Germany), according to the manufacturer’s instructions. The purified RT-PCR products were subjected to Sanger sequencing using the ABI PRISM BigDye terminator cycle sequencing kit (PerkinElmer, Foster City, CA, USA).

#### 2.2.5. Phylogenetic Analysis

The nucleotide sequence and deduced amino acid sequences of these H9 isolates were blasted and compared with the reference strain sequences retrieved from GenBank from different regions of the world. Bioedit 7.2.5 software [19] was used to compare and align nucleotide sequences. Phylogenetic analysis and tree construction for the HA gene were generated using the maximum likelihood (ML) method, with MEGA software Version 5.05 program with the Tamura-Nei model [20].

## 3. Results

### 3.1. Case History and Pathological Findings

#### 3.1.1. Case History and Seasonal Distribution of Outbreaks

**Figure 1:**
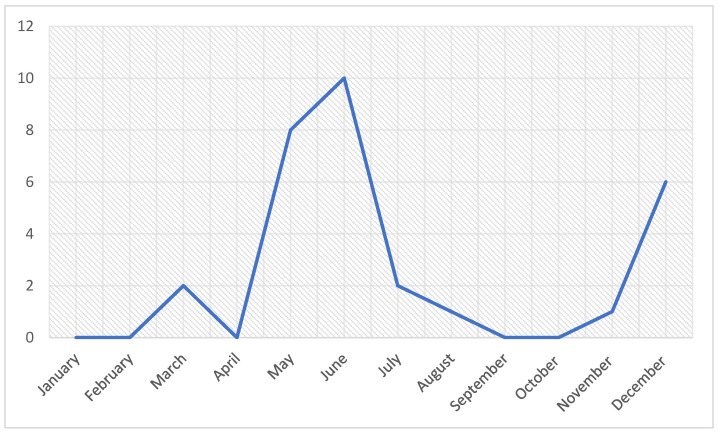
Temporal distribution of LPAI H9N2 outbreaks in houbara bustards and gamebirds in Morocco.

For this study, a total of 93 specimens from suspected birds were investigated based on clinical signs and gross pathologic lesions. The birds were submitted to the clinic because of respiratory symptoms and mortalities (Table A1). As for the seasonal distribution, 70% of cases were recorded between May and August with another 20% recorded during the month of December (Figure 1).

#### 3.1.2. Gross Pathological Findings

**Figure 2:**
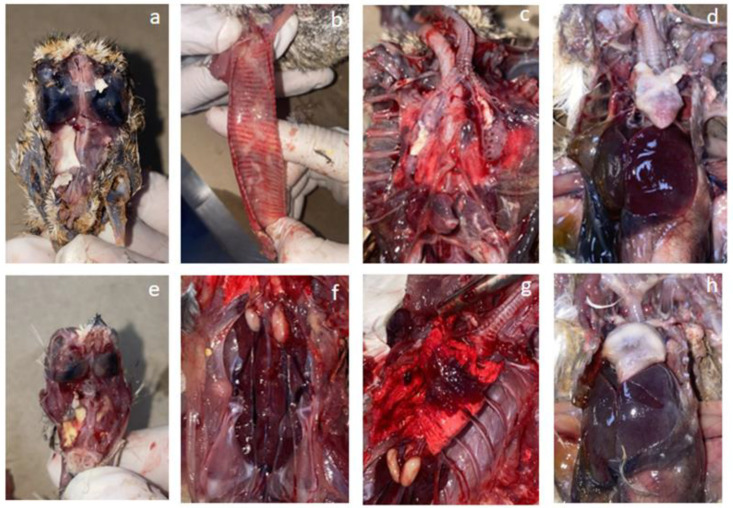
Gross pathological lesions associated with LPAI H9N2 infection in houbara bustards and gamebirds. Photos (**a**–**d**) represent pathological lesions observed in H9N2-infected bustards: (**a**) fibrinous sinusitis, (**b**) congestive and exudative tracheitis, (**c**) intrabronchial fibrin cast, (**d**) fibrinous pericarditis with liver congestion. Photos (**e**–**h**) represent lesions observed in gamebirds: (**e**) fibrinous sinusitis in a Barbary partridge, (**f**) kidney congestion and hypertrophy in a Barbary partridge, (**g**) lung congestion in a Japanese quail, (**h**) fibrinous pericarditis and perihepatitis in a Barbary partridge.

Necropsy of the LPAI H9N2 positive cases revealed a variety of lesions depending on the coinfecting agents; however, the main consistent lesions were fibrinous sinusitis (30%) and tracheitis in 20% (6/30), and 43.3% (13/30) for both congestive lungs with fibrin plugs in the bronchial lumen and airsacculitis (Figure 2). Other recurring lesions included fibrinous pericarditis and perihepatitis in the context of bacterial coinfections in 43.3% (13/30). Catarrhal enteritis, hypertrophy and congestion of the kidneys, and splenomegaly were noticed in 70%, 43.3%, and 13.67% of cases, respectively. On the other hand, pancreas hypertrophy was detected in only 5/30 (16.67%) of cases, all of which involved bustards with a septicemic poxvirus infection.

#### 3.1.3. Histopathological Lesions

**Figure 3:**
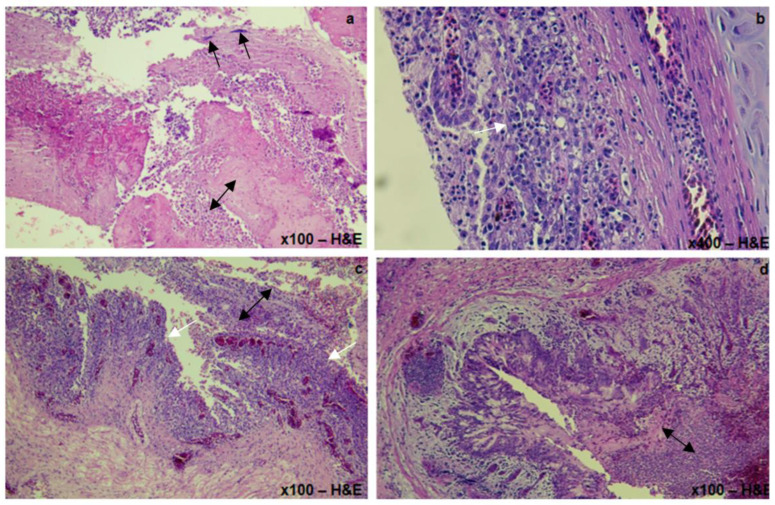
Histopathological lesions of the trachea and lungs in a confirmed LPAI H9N2 infection in a houbara bustard with coinfection with *E. coli* associated to Staphylococcus Aureus: (**a**) Trachea. Fibrino-heterophilic material accumulated in the tracheal lumen (double-headed arrow); note the intralesional dense bacterial colonies (black arrow), (**b**) Trachea. Congestion of blood vessels with lympho-plasmocytic and heterophilic infiltration in the lamina propria (white arrow), (**c**,**d**) Lung. Severe congestion in the bronchial mucosa associated with lymphocytic and heterophilic infiltration (white arrow) and accumulation of cellular and fibrino-necrotic material in the bronchial lumen (double-headed arrow).

Histopathology revealed extensive fibrino-heterophilic and lymphocytic tracheitis and airsacculitis, multifocal to extensive lympho-plasmocytic, and heterophilic bronchopneumonia, with intralesional bacterial colonies (Figure 3). Degenerative changes were noticed in the tubular epithelium of the kidneys and multifocal depletion of zymogen granules of the pancreas were observed. A severely extensive, necrotizing, heterophilic pancreatitis with eosinophilic intracytoplasmic inclusions was noticed in all cases with poxvirus infection.

### 3.2. H9N2 Identification and Phylogenetic Analysis

#### 3.2.1. H9 Detection by Real Time RT-PCR

A total of 30/93 (32.26%) samples were positive for H9N2, comprising 23/69 houbara bustards, 4/13 partridges, 2/3 quails, and 1/4 pheasants, with Cts ranging from 22.3 to 38.3 (Table A1). The age of the affected birds varied from 15 days to 105 weeks irrespective of their H9N2 vaccination status. As for the house sparrows, 3/10 (30%) tested positive for LPAI H9N2 virus.

#### 3.2.2. Virus Isolation and the Partial HA Gene Amplification

All the samples found to be positive for LPAIV H9N2 using real-time RT-PCR (*n* = 30) were subjected to conventional RT-PCR. Isolation in SPF eggs was attempted for ten samples with a lower viral load (Ct values greater than 35) in order to obtain a maximum viral load detectable by conventional RT-PCR and for HA gene sequencing purposes. Conventional RT-PCR analysis revealed that HA PCR products could be obtained only for two isolates derived from partridges. The nucleotides sequences of both characterized H9N2 isolates were submitted to the GenBank database under accession numbers OR293335 and OR293336.

#### 3.2.3. BLAST Search and Phylogenetic Analyses

**Figure 4:**
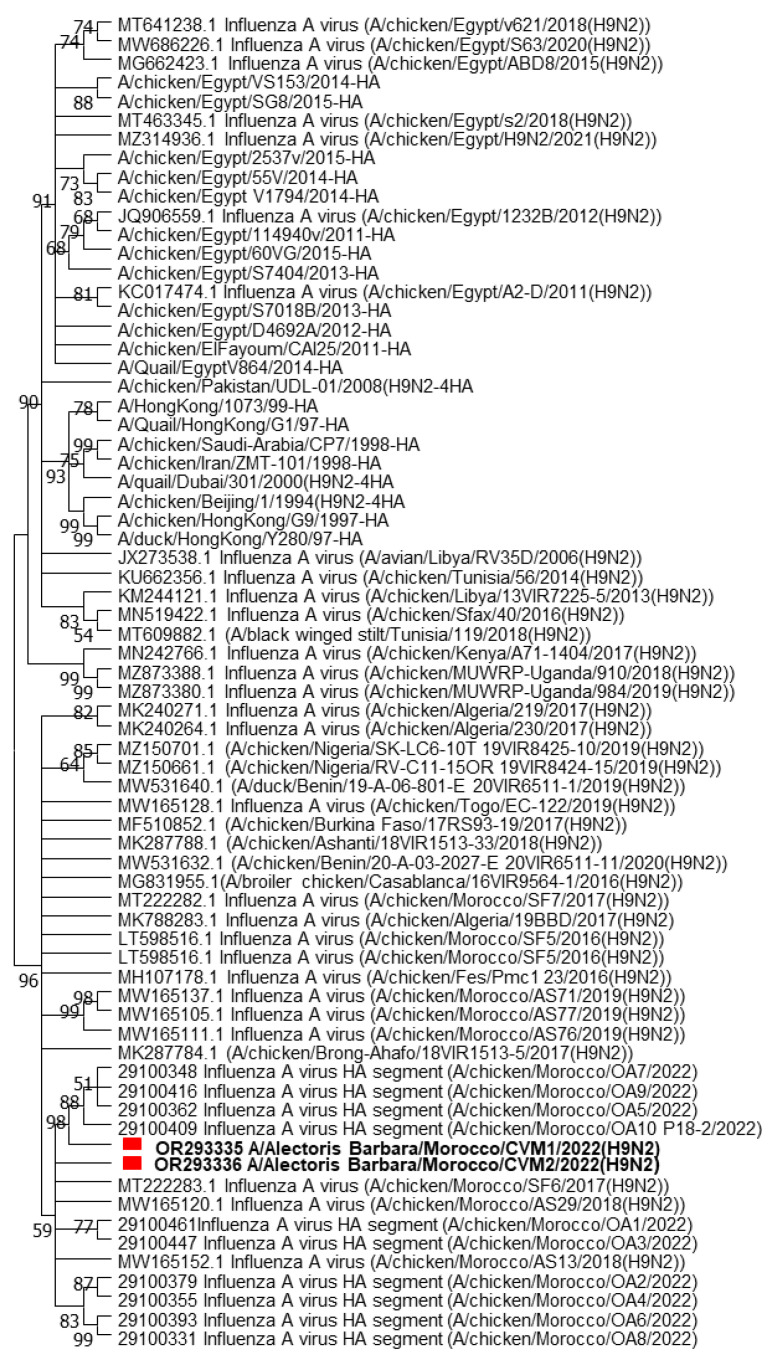
Phylogenetic tree of the Moroccan partridge isolates. The nucleotide sequences of Moroccan H9N2 viruses (red squares) characterized in this study were compared with seventy relevant virus sequences available in GenBank and GISAID databases, reference viruses, and relevant sequences from neighboring areas. The length of the HA gene sequences used in the phylogeny is 500 pb.

The nucleotide and amino acid sequences of the Moroccan strains were highly similar and presented a 97.33% nucleotide sequence identity with A/chicken/Algeria/17BBD/2017 and A/chicken/Morocco/SF4/2016, H9N2 viruses detected in broiler chickens in Algeria and Morocco in 2017 and 2016, respectively. Both Moroccan isolates had the RSSR*GLF motif at the HA cleavage site, which is a characteristic and signature of the low pathogenic H9N2 viruses. Based on the HA phylogenetic tree, both Moroccan partridge isolates were closely related to viruses previously isolated in Morocco in 2017 and 2022 with a bootstrap value of 98 (Figure 4) and classified in the same cluster as viruses isolated from Algeria (2017), Burkina Faso (2017), Nigeria (2019), and Togo (2020). They all belonged to the G1 lineage or Lineage A, based on the recent classification [21]. All the viruses were closely related to each other.

### 3.3. Differential Bacteriological and Molecular Diagnosis

Coinfection with *E. coli* was noticed in 60% (18/30) of the cases. In addition, *Staphylococcus Aureus*, *Pseudomans Aeruginosa* and *Enterococcus* sp. were also recurring complicating bacterial pathogens with a distribution of 13.33% (4/30) for each. As for fungal coinfecting agents, five bustards (16.67%) were positive for *Candida Albicans*, and two other cases (6.67%) had a confection with *Aspergillus Fumigatus*, one of which was a quail and the other a partridge (Table 1). Conversely, all the samples tested negative for other viral pathogens including NDV, ILT, AMPV, and *Chlamydia psittaci*. However, one pheasant tested positive for MG associated with H9N2, three bustards tested positive for both H9N2 and avian coronavirus, and two tested positive for coronavirus only.

As for the house sparrows, a total of 8/10 (80%) tested positive for avian coronavirus, yet they all tested negative for *Mycoplasma* sp. and *Trichomonas* sp. Furthermore, the sparrows tested positive for *E. coli*, *Klebsiella* sp., *Staphylococcus Aureus*, and *Enterococcus* sp. A scraping of the mucosal surface of the intestines revealed the presence of *Eimeria* sp., although the species could not be identified. Additionally, *Tetrameres* sp. was present in the proventriculi of three birds (Table 1).

## 4. Discussion

We report in this article the detection of the LPAI avian influenza subtype H9N2 in diseased houbara bustards and gamebirds in Morocco including pheasants, quails, and partridges. During the study period, the most affected species were houbara bustards, with 23 positives among 69 tested birds (33.33%), followed by partridges, then quails, and lastly pheasants, with 4/13 (30.7%), 2/3 (66.67%) and ¼ 1/ 4 (25%) positives for each species, respectively.

Houbara bustards are susceptible to infection by avian influenza viruses; in fact, both highly pathogenic and low pathogenic avian influenza were previously reported in diseased Asian bustards in the United Arab Emirates and Saudi Arabia [22,23]. In addition, Wernery et al. [24] demonstrated the susceptibility of these species through an experimental infection using an LPAI H9N2 strain in two houbara bustards, following which the authors reported clinical signs and pathological lesions similar to those observed in field outbreaks of H9N2 infection in chickens and turkeys. Moreover, a recent serological survey of antibodies against H9, H7, and H5 avian influenza viruses in falcons and other wild bird species including houbara bustards and white-bellied bustards revealed a seroprevalence of 12.1% (16/132) and 15% (3/20) positives for antibodies against H9 in these birds, respectively. Conversely, other avian influenza subtypes were negative, which demonstrates the wide distribution and circulation of AIV H9 viruses among wild birds of the UAE, including bustards [25].

On the other hand, several studies on LPAIV H9N2 in gamebirds have confirmed the susceptibility of these species to the virus [26,27], contrary to its pathogenicity, which is not fully substantiated, notably in quails and pheasants [13]. In fact, some reports indicate an asymptomatic carriage [13,27,28] both in field and experimental settings, while others describe symptomatic infection with respiratory clinical signs and a drop in egg production of up to 30% specifically in quails [13,29,30,31]. Owing to their important epidemiological role as intermediate hosts in avian influenza virus transmission and adaptation to mammals, these species have been the focus of extensive studies on AIV replication [32]. Indeed, it was proven that these species, along with partridges, possess both α-2,3 and α-2,6 receptors in the respiratory and intestinal tracts, which makes them a potential source of avian influenza viruses with pandemic potential [32,33]. In addition, studies have proven pathogenicity to be different among these species; indeed, Humberd et al. [14] demonstrated the low susceptibility of ring-necked pheasants to avian influenza viruses, and the authors suggested the capacity of pheasants to serve as reservoirs considering the long period of viral shedding (up to 14 days post-infection) in an asymptomatic fashion. Moreover, Świętoń et al. [28] reported the wide heterogeneity of avian influenza viruses in the oropharynx of an experimentally infected bobwhite quail, along with high viral shedding and an asymptomatic course of infection, which further demonstrates the role of quails as an intermediate host for the adaptation of AIV to domestic poultry. These studies correlate with our findings, since over a 3-year period we have received only three cases of quails and four others from flocks of pheasants with respiratory signs involving H9N2 suspicion. Still, our study was based on clinical cases submitted for diagnosis; therefore, due to sampling bias, the interpretation of these findings may be biased as well. On the other hand, studies of the seroprevalence along with molecular detection of the virus on filed samples can give a better idea of the prevalence of LPAIV H9N2 in Moroccan gamebirds. Similarly, the cases that we report herein exhibited a seasonal pattern, since the submitted cases were concentrated between May and June. Although similar findings were reported by Kent et al. [22], the seasonal distribution and the prevalence of H9N2 infection cannot be confirmed unless studied with the help of appropriate statistical methods on appropriately selected field samples.

As for partridges, the pathogenicity of the virus was confirmed in Chukar partridges (*Alectoris chukar*) and red-legged partridges (*Alectoris rufa*) [13,31,34,35,36,37,38], causing severe respiratory signs with low to zero mortality rates. As for Barbara partridges (*Alectoris barbary*), there are currently no data on the infection. Our study represents the first report of a field outbreak in these species worldwide. Still, the evaluation of the susceptibility and pathogenesis of LPAI H9N2 in Moroccan Barbary partridges is advisable in order to understand the pathobiology of the virus in these species, since striking species–specific differences in susceptibility and in the pathogenicity of the virus were previously reported by Jöstl et al. [25].

We report in these natural outbreaks of H9N2 in bustards and gamebirds severe respiratory distress with elevated mortality rates. These findings correlate with [24], who conducted an experimental infection with LPAIV H9N2 in houbara bustards and reported severe respiratory clinical signs with dyspnea, lethargy, and anorexia followed by death. On the pathological level, the authors reported similar findings to those we found in the outbreaks, except for pancreatitis, which we found only in bustards with a systemic poxvirus infection confirmed by histopathology. Although the authors reported these lesions in the experimental infection using the H9N2 virus alone, it is most likely that the virus was associated to other bacterial pathogens, since the virus alone cannot induce these lesions, which was confirmed in specific pathogen-free chickens (*Gallus Gallus*) [39,40,41]. In the outbreaks that we report, in 83.33% (25/30) of the cases, LPAI was associated with bacterial agents. Furthermore, in 36,67% (11/30) of cases, one complicating agent was identified, often *E. coli*, and in 43.3% of cases (13/30), the virus was associated with multiple other bacterial, fungal, and viral respiratory pathogens as follows: 30% (9/30) with double coinfections, 13.33% (4/30) with triple coinfections, and 3.33% (1/30) with quadruple coinfections. In addition, clinical signs, mortality rates, and pathological lesions were more severe in the outbreaks involving multiple coinfecting agents. Similar findings were reported by [42] in a survey of broiler flocks in Pakistan. The synergetic effect of LPAI H9N2 with other pathogens, namely, *Mycoplasma Gallisepticum*, has been demonstrated, first by their close association in severe clinical field outbreaks in broilers [39,41,43], and in experimental settings [40,44]. Coinfection with avian coronavirus is another cause of the exacerbation of LPAIV H9N2 infection in chickens [45,46,47,48]. However, the presence of an avian coronavirus in the houbara bustards in our study is probably of no pathological significance, although its pathogenesis in these species cannot be excluded [49]. The only reports of the presence of coronaviruses in bustards were deltacoronaviruses detected sporadically in the molecular surveillance of diseases, with no pathological or clinical impact on birds [50,51,52]. The presence of the virus in these species in association with their predators, mainly falcons, was linked to the food chain [50]. As for our findings, the virus is likely transmitted to bustards from house sparrows.

As for the origin of LPAIV H9N2, in the case of the rearing units located in the regions of Tangier, Rabat, and Casablanca, which house mainly gamebirds (cases 4, 17, 22, and 29 in Table A1), there is a considerable risk of virus transmission from the poultry farms present in these regions, since they are considered hotspots of avian infectious diseases due to the high poultry farm density in the area (Mouahid, M., personal communication, 2023). Gamebirds, on the other hand, can harbor the virus and spread it reciprocally to other intensive poultry farms [34], which further enhances viral load and persistence in these regions. As for other regions located in southern Morocco, the virus origin remains unclear. The first theory, which involves transmission by waterfowl (*Anseriformes* order) and shorebirds (*Charadriiformes* order), which are considered a reservoir of AIV [53,54], cannot be confirmed, since birds of these orders are not common in the said regions. Furthermore, [11] reported a prevalence of only 1.86% (18/976 samples) amongst wild birds, mostly *Charadriiformes*. For *Anserifomes*, however, the PCR for H9N2 was negative for all the sampled specimens; similarly, [25] reported negative antibody titers against all the tested subtypes (H9, H7, and H5) in mallards in the UAE, which corroborates the results of El Mellouli et al. (2022) [11]. Additionally, the environmental conditions of the regions, including high temperatures and ultraviolet indexes and low humidity, do not support AIV survival and persistence [25,55]. This means that the transmission of the virus to houbara bustards might have involved birds from other taxa [55]. In fact, we found 30% (3/10) positive specimens derived from house sparrows and it is possible that the outbreaks were caused by contact with these birds. Notwithstanding, the opposite scenario cannot be ruled out, which means contamination of house sparrows from diseased houbara bustards, since aviaries can offer an ideal opportunity for viral transmission in both directions through repeated contacts between birds of both species [55].

In our study, among 30 positive samples obtained by real time RT-PCR, only two AIV could be sequenced. The difficulty of amplifying the AIV genome in wild birds has already been reported by several studies. Kim and coauthors [56] noticed differences in molecular test results including RT-PCR, conventional RT-PCR, and virus isolation. In fact, they tested 11,145 fecal samples of wild birds and reported 50 positives using virus isolation; among these, only 52% tested positive using RT-PCR. These discrepancies are explained by differences in primer sequences and a lack of validation in wild bird species, which lead to lower sensitivity [57,58]. In addition, the specificity of the RT-PCR can also be lower for specimens originating from wild birds [59].

During this study, the sequencing of viruses derived from partridges showed a 97.33% nucleotide identity with the strains isolated previously in broilers in Morocco in 2017 and 2022 [10]. On the HA phylogeny, the AIV sequences from the partridges were close to those from Morocco (2022), Algeria (2017), Burkina Faso (2017), and Togo (2020). This similarity can be explained by the common border between the two countries and by the history of commercial exchanges within western African countries. In 2016, the first identification of LPAI H9N2 viruses in Morocco was reported. In addition, Moroccan isolates showed over 99% similarity and formed a distinct cluster with isolates of pheasants and whit-bellied bustards from the UAE in 2011. and a distinct cluster was formed with isolates of pheasants and white-bellied bustards from the UAE in 2011 [7].

## 5. Conclusions

In conclusion, LPAIV H9N2 is circulating in houbara bustards and gamebirds (quails, partridges, and pheasants) in Morocco, and house sparrows could be a possible source of infection. Further studies need to be carried out in order to understand the transmission dynamics between these species and other wild and reared birds, including falcons, since houbara bustards are used as their primary quarry. In addition, we think that studies on the assessment of vaccination as a means of virus spread control in these species is advisable. We also recommend further active surveillance of AIV and avian coronaviruses in these species since they can constitute a prominent risk factor for the perpetuation of the circulation of the virus and the enhancement of mutations leading to the emergence of new genotypes.

## Figures and Tables

**Table 1:** Results of the molecular, bacterial, and parasitological screening of house sparrows sampled from the premises of diseased houbara bustards.

House Sparrows Sample Number	H9N2 PCR Results (Ct)	Other Viral Pathogens	Parasitic Pathogens	Bacterial Pathogens *
1	37.5	N	*Tetrameres* sp. *Eimeria* sp.	*E. coli* *Klebsiella* sp. *Staphylococcus Aureus* *Enterococcus* sp.
2	38.6	Avian coronavirus	*Tetrameres* sp.	
3	N	Avian coronavirus	*Eimeria* sp.	
4	N	N	*Tetrameres* sp.	
5	37.5	Avian coronavirus	N	
6	N	Avian coronavirus	*Eimeria* sp.	
7	N	Avian coronavirus	*Eimeria* sp.	
8	N	Avian coronavirus	N	
9	N	Avian coronavirus	*Eimeria* sp.	
10	N	Avian coronavirus	N	

N: Negative; *: pooled organs were taken for bacteriology.

## Data Availability

Data are contained within the article.

## Appendix A

**Table A1:** Case history of LPAI H9N2 confirmed outbreaks in gamebirds and in Houbara bustards of Morocco.

Cases	Bird Species	Bird Population	Age (d/w)	H9 Vaccinationstatus (V/NV)	RT-PCR H9 (Ct)	Clinical Signs	Mortality Rate (%)	Pathological Lesions	Coinfecting Agents
1	Quail	ND	ND	NV	38.34	Mild rales	None	conjoncitivitis	Negative
2	Pheasant	ND	ND	NV	37.47	Rales, Head swelling	None	Fibrinous sinusitis; catarrhal enteritis	*Mycoplasma Gallisepticum*
3	Houbarabustard	ND	8 w	NV	33.97	Coughing, tracheal rales, respiratory distress, anorexia,	NA	Purulent arthritis; Airsacculitis; fibrinous pericarditis; hepatomegaly; splenomegaly; catarrhal enteritis; kidney congestion and hypertrophy; congested lungs	*E.Coli* *Enterococcussp*
4	Partridge	23,000	3 w	V	32.72	Tracheal rales, diarrhea, lethargy	0.33%	Fibrinous sinusitis; fibrinous tracheitis; airsacculitis; kidney hypertrophy; catarrhal enteritis	*E.Coli*
5	Houbarabustard	ND	200 d	NV	36.69	Coughing, sneezing, tracheal rales, dyspnea, diarrhea	NA	Purulent sinusitis; fibrinous tracheitis; fibrinous airsacculitis and perihepatitis; enteritis; ulcerative stomatitis	*E.Coli* *Staphylococcus Aureus* *Pseudomonas sp* *Candida Alibcans*
6	Houbarabustard	ND	33 d	NV	25.89	Coughing, sneezing, tracheal rales, dyspnea, diarrhea	NA	Intrabronchial fibrin cast; airsacculitis; fibrinous pericarditis and perihepatitis; catarrhal enteritis	Avian Coronavirus *E.Coli* *Enterococcussp*
7	Houbarabustard	ND	25 d	NV	37.69	Coughing, sneezing, tracheal rales, dyspnea, diarrhea, lethargy	NA	airsacculitis; fibrinous pericarditis and perihepatitis; catarrhal enteritis; kidney hypertrophy and congestion	*E.Coli* *Pseudomonas Aerogenosa* *Enterococcussp*
8	Houbarabustard	ND	17 d	V	26.79	Coughing, sneezing, tracheal rales, dyspnea, diarrhea	NA	Congestive lungs; airsacculitis; fibrinous pericarditis and perihepatitis; catarrhal enteritis; kidney hypertrophy and congestion	*E.Coli*
9	Houbarabustard	ND	36 d	V	23.54	Coughing, sneezing, tracheal rales, dyspnea, diarrhea, lethargy	NA	Intrabronchial fibrin cast; fibrinous airsacculitis; catarrhal enteritis Congestive liver	Avian coronavirus *E.coli* *Pseudomonas Aeroginosa*
10	Houbarabustard	ND	63 d	V	22.32	Coughing, sneezing, tracheal rales	NA	Congestive lungs; fibrinous airsacculitis; catarrhal enteritis congestive liver	Avian coronavirus *E.Coli*
11	Houbarabustard	ND	26 d	V	34.7	Tracheal rales, coughing	NA	tracheal congestion; lung congestion; liver congestion; thickened air sacs	Negative
12	Houbarabustard	ND	33 d	V	26.27	Tracheal rales, coughing	NA	Tracheal congestion; lung congestion; liver congestion; thickened air sacs	Negative
13	Houbarabustard	ND	43 d	V	30.31	Tracheal rales, coughing	NA	Tracheal congestion; lung congestion; liver congestion; thickened air sacs	Negative
14	Houbarabustard	ND	71 d	V	33.1	Tracheal rales, coughing	NA	Tracheal congestion; lung congestion; liver congestion; thickened air sacs	Negative
15	Houbarabustard	ND	76 d	V	36.94	Coughing, sneezing, tracheal rales, dyspnea	NA	Fibrrinous sinusitis; lung congestion; fibrinous pericarditis; fibrinous airsacculitis	*E.Coli* *Enterococcussp.*
16	Houbarabustard	ND	55 d	V	36.36	Coughing, sneezing, tracheal rales	NA	Lung congestion; fibrinous pericarditis; fibrinous airsacculitis	*E.Coli*
17 *	Partridge	9365	32 w	V	26.19 *	Tracheal rales, diarrhea, cachexia	0.5%	Fibrinous sinusitis and congestive tracheitis; Intrabronchial fibrin cast; fibrinous pericarditis and perihepatitis; splenomegaly	*E.Coli*
18	Houbarabustard	ND	201 d	V	35.17	Dyspnea, diarrhea, anorexia, lethargy	NA	Exsudativetracheitis with intrabronchial fibrin cast; airsacculitis; perihepatitis with hepatomegaly; splenomegaly; catarrhal enteritis; ulcerative oesophagitis, pancreas hypertrophy	Poxvirus *MycoplasmaGalliseptcium* *Candida Albicans*
19	Houbarabustard	ND	237 d	V	38.32	Diarrhea, anorexia, diarrhea, lethargy	NA	Purulent arthritis; aisacculitis; hepatomegaly with perihepatitis; ulcerative esophagitis; catarrhal enteritis; pancreas hypertrophy	Poxvirus *E.Coli* *Staphylococcus sp* *Candida Albicans*
20	Houbarabustard	ND	235 d	V	36.27	Diarrhea, anorexia	NA	Exsudative tracheitis; fibrinous pericarditis and perihepatitis with hepatomegaly; catarrhal enteritis; pancreas hypertrophy; ulcerative oesophagitis and stomatitis, pancreas hypertrophy	Poxvirus *Staphylococcus Aureus* *Candida Albicans*
21	Houbarabustard	ND	220 d	V	37.34	Diarrhea, anorexia	NA	Airsacculitis; fibrinous pericarditis and perihepatitis with hepatomegaly; catarrhal enteritis; kidneyshy pertrophy and congestion; pancreashy pertrophy	*Candida Albicans*
22 *	Partridge	4000	105 w	V	35.23	Head swelling, tracheal rales, dyspnea, paralysis, overcrowding, diarrhea	0.5%	Fibrinous sinusitis; fibrinous otitis media and interna; intrabronchial fibrin cast; catarrhal enteritis; kidney hypertrophy and congestion	*E.Coli* serotype O2K1 *Aspergillus Fumigatus*
23	Houbarabustard	ND	35 w	V	30.54	Coughing, tracheal rales, dyspnea, diarrhea, anorexia	NA	Intrabronchial fibrin cast; airsacculitis; fibrinous pericarditis and perihepatitis; enteritis; kidney hypertrophy; ulcerative stomatitis and stomatitis, pancreas hypertrophy	Poxvirus *E.Coli*
24	Houbarabustard	ND	35 w	V	27.16	Coughing, sneezing, tracheal rales, dyspnea, diarrhea, anorexia	NA	Intrabronchial fibrin cast; airsacculitis; fibrinous pericarditis and perihepatitis; enteritis; kidney hypertrophy and congestion; ulcerative stomatitis, pancreas hypertrophy	Poxvirus *Klebsiellasp*
25	Houbarabustard	2500	15 d	V	32.1	Coughing, sneezing, tracheal rales, dyspnea, diarrhea	NA	Fibrinous sinusitis; intrabronchial fibrin cast; airsacculitis; fibrinous pericarditis; liver congestion; enteritis; kidney hypertrophy and congestion	*E.Coli* *Enterococcussp*
26	Houbarabustard	ND	26 d	V	33.26	Coughing, tracheal rales, dyspnea, diarrhea	NA	Fibrinous sinusitis; intrabronchial fibrin cast; fibrinous airsacculitis; enteritis; kidney congestion and hypertrophy	*E.Coli*
27	Houbarabustard	ND	38 d	V	31.45	Coughing, tracheal rales, dyspnea, diarrhea	NA	Intrabronchial fibrin cast; liver congestion; enteritis; kidney congestion hypertrophy	*E.Coli*
28	Houbarabustard	ND	23 d	V	37.19	Coughing, tracheal rales, dyspnea, diarrhea	NA	Fibrinous sinusitis; intrabronchial fibrin cast; airsacculitis; pericarditis; enteritis; kidney hypertrophy and congestion	*E.coli*
29	Partridge	18,600	2 w	V	33.21	Dyspnea, rales, diarrhea, lethargy	0.3%	Fibrinous sinusitis; lung congestion with nodules; fibrinous pericarditis; kidney congestion; enteirits	*E. Coli* *Aspergillus Fumigatus*
30	Quail	ND	ND	V	33.6	Respiratory distress	<0.01%	Lung congestion; airsacculitis; liver congestion; kidney congestion and hypertrophy	*Mycoplasma Galliseptcium* *Aspergillus Fumigatus*

ND = No Data; *: sequenced H9N2 isolates. V= vaccinated at 1 day-old using an inactivated vaccine via intra-muscular route; NV = not vaccinated; NA: Not applicable, mortality rates in bustards cannot be calculated since each bird is examined separately.
